# A pH-sensitive film based on chitosan/gelatin and anthocyanin from *Zingiber striolatum* Diels for monitoring fish freshness

**DOI:** 10.1016/j.fochx.2024.101639

**Published:** 2024-07-16

**Authors:** Yuyue Qin, Yurou Wang, Zhenya Tang, Kejun Chen, Zhengxuan Wang, Guiguang Cheng, Hai Chi, Thanapop Soteyome

**Affiliations:** a Faculty of Food Science and Engineering, Kunming University of Science and Technology, Kunming 650550, China; b Faculty of Modern Agricultural Engineering, Kunming University of Science and Technology, Kunming, 650550, China; c Yunnan International Joint Laboratory of Green Food Processing, Kunming 650500, China; d College of Food and Bioengineering, Xihua University, Chengdu 610039, China; e Rajamangala University of Technology Phra Nakhon, Bangkok 10300, Thailand

**Keywords:** Anthocyanin, Fish freshness, pH-sensitive film, Visible color change, *Zingiber striolatum* Diels

## Abstract

As a new type of packaging method, the anthocyanin-based pH-sensitive indicator film has gained much attention owing to low cost, small size, and visually informative property. In this study, an intelligent film based on chitosan/gelatin (CG) matrix with *Zingiber striolatum* Diels (ZSD) anthocyanin for fish freshness monitoring was developed. The film properties, including thickness, moisture content, color, mechanical properties, UV–vis light barrier property, as well as pH and ammonia sensitivity, were evaluated. The CG-ZSD films exhibited a more compact structure when compared with the CG film. The CG-ZSD20 film showed the highest elongation at break (6.33 ± 0.62%) and lowest tensile strength (20.0 ± 0.58 MPa). FTIR spectra revealed the strong hydrogen bond interactions between ZSD and polymer matrix. Film incorporated with 15% anthocyanin extract has increased melting temperature at 118.9 °C, and a lower weight loss (13.8%) at melting temperature. In pH 1–14 buffer, the color of CG-ZSD films underwent a significant change from red to yellow-green. The CG-ZSD15 film was utilized for monitoring fish freshness and showed visible color changes from deep purple to brown. The total volatile basic nitrogen content and pH value changes of fish were closely related to the visual color changes in film. This demonstrated that the film was a highly pH-sensitive film for quantifying fish freshness in real-time.

## Highlights

• The indicator film was prepared by blending chitosan with gelatin.
• The pH-sensitive film was based on anthocyanin from *Zingiber striolatum Diels.*
• Effect of *Zingiber striolatum Diels* on the physicochemical property was determined.
• The infrared spectra confirmed the structural change in matrix and anthocyanins.
• The film was applied to indicate fish freshness with evident color change.

## 1 Introduction

The pH-sensitive film is an innovative type of packaging material developed recently for the detection of food decomposition or freshness, with anthocyanin being used as the indicator component. Anthocyanin is a water-soluble phenolic compound. The structure of phenolic or conjugated compounds, such as cyanidin, delphinidin, and pelargonidin in anthocyanin, varies with pH, leading to changes in the color of anthocyanin (Pourjavaher et al., 2017).

Meat product would undergo deterioration due to microbiological, chemical, and physical factors. Measuring pH value and ammonia concentration of meat product is one approach to identify its deterioration process. Extensive researches have been conducted on incorporating anthocyanins from mulberry pomace (Zhang et al., 2021), red cabbage (Pourjavaher et al., 2017), black peanut seed coat (Lu et al., 2022), raspberry (Duan et al., 2022), black soybean seed coat (Wang et al., 2019) and butterfly pea flower (Li et al., 2024) to prepare colorimetric indicator films for monitoring food quality.

*Zingiber striolatum* Diels (ZSD), a perennial herb belonging to the Zingiberaceae family and Zingiber genus (Liu et al., 2018), is commonly known as ‘Yanghe’ in China. The inflorescences of ZSD have a purplish-red color and are a rich source of anthocyanin. Previous research has identified flavonoids, peonidin-3-rhamnoside and malvidin-3-galactoside as the primary constituents of the red pigment in ZSD (Liu et al., 2023). However, no studies have investigated the application of ZSD red pigment in pH-sensitive film. The young and tender inflorescences of ZSD are usually used to be cooked, and the hard and aging layer is often discarded. Preparing the indicator film with the underutilized part is also a recycling of ZSD resources.

Chitosan is a polysaccharide obtained through the deacetylation of chitin (Lu et al., 2022). It consists of D-glucosamine and *N*-acetyl-D-glucosamine units (Duan et al., 2022). Chitosan is a biodegradable and non-toxic polysaccharide with notable antibacterial, film-forming, and biodegradable properties. However, pure chitosan films lack strong antibacterial and antioxidant capabilities. To address this, the functional property of chitosan films could be enhanced by incorporating natural products such as plant extracts, essential oils, bacteriocins, and phenolic compounds, which possess antioxidant or antibacterial activity. It has been demonstrated that the addition of a specific amount of anthocyanin to chitosan films helps improve their mechanical strength, water vapor resistance, and UV–vis light barrier properties (Wang et al., 2019).

Gelatin is a biopolymer derived from the hydrolysis of animal collagen (Li et al., 2024). Pure gelatin film exhibits low water resistance, weak ductility, and limited solubility in water. However, it possesses several advantages, including strong biocompatibility, high transparency, and high hardness (Li et al., 2023). To enhance the functional properties of gelatin films, phenolic compounds derived from plants, including anthocyanins, can be employed (Wang et al., 2019). Some studies have been reported that the chitosan/gelatin blend could provide alternative polymer matrix for indicator films, with better mechanical property and lower swelling property (Lu et al., 2022).

In this paper, pH-sensitive films based on chitosan, gelatin, and anthocyanin from ZSD were prepared to indicate fish freshness. The content of ZSD anthocyanin, as well as film thickness, moisture content, mechanical properties, color parameters, and pH sensitivity were evaluated. Additionally, we analyzed the microstructure of the films using Fourier transform infrared and scanning electron microscopy.

## 2 Materials and methods

### 2.1 Materials

Chitosan (Mw = 150 KDa, Deacetylation degree = 75%) was obtained from Henan Jiazhi Biotechnology Co., Ltd. (Zhengzhou, China). Gelatin (Type B from bovine skin, biochemical reagent) was obtained from Fuchen Co., Ltd. (Tianjin, China). *Zingiber striolatum* Diels was sourced from Kunming, China. All chemicals used in this study were of analytical grade purity. *Pelteobagrus fulvidraco* was obtained from Yonghui Supermarket, Kunming, China. The fish was slaughtered and delivered to the laboratory within 30 min.

### 2.2 Extraction of ZSD anthocyanin

Anthocyanin was extracted from ZSD following the procedure described by Chen et al. (2023). ZSD was washed, dried, and pulverized. Subsequently, 20 g of ZSD powder was mixed with 400 mL of 75% (*v*/v) ethanol aqueous solution, using a material-liquid ratio of 1:20 (g/mL). The result solution was performed by ultra-sonication for 30 min at room temperature, avoiding sunlight exposure. The excess ethanol solution was then evaporated at 50 °C. The result concentrate was further lyophilized to obtain ZSD anthocyanin powder.

### 2.3 UV–vis spectra of ZSD extract

The buffer solution ranging from pH 1 to 14 was prepared by adjusting with 0.1 mol/L of HCl solution or 1 mol/L of NaOH solution. About 50 μL of ZSD extract was added into 5 mL of buffer solution. Photographs were taken to document the color change of ZSD extract solution, and the spectrogram was determined using a dual-beam UV–vis spectrophotometer (T9CS, Beijing Persee General Instrument Co., Ltd., Beijing, China) within the range of 450–700 nm (Akhila et al., 2023).

The L, a, and b value of ZSD extract were determined at pH 1–14, using a WSC-S Chroma Meter with D6 as the light source. The color difference (∆E) was calculated by formula (1):

(1) $$∆E=\sqrt[{2}]{{\left(L^{*}-L\right)}^{2}+{\left(a^{*}-a\right)}^{2}+{\left(b^{*}-b\right)}^{2}}\;$$

where L * (91.29), a * (−0.89), and b * (3.76) represent the color of white plate used for calibration.

### 2.4 Quantification of ZSD anthocyanin

The content of ZSD anthocyanin was qualified following the procedure described by Anugrah et al. (2023). The ZSD anthocyanin powder was diluted 10-fold with KCl buffer (pH 1.0) and NaOAc buffer (pH 4.5), respectively. After equilibrating in the dark for 15 min, the absorbance value at 520 nm and 700 nm were measured. The content of ZSD anthocyanin was determined using (2), (3):

(2) $$\begin{array}{c} A={\left[\left(A_{520}-A_{700}\right)\right]}_{\mathrm{pH}1.0}-{\left[\left(A_{520}-A_{700}\right)\right]}_{\mathrm{pH}4.5} \end{array}$$

(3) $$\begin{array}{c} C=\left(\mathrm{mg}/L\right)=\frac{A\times 449.2\times \mathrm{DF}\times 1000}{\left(\varepsilon \times L\right)} \end{array}$$

where C is the content of anthocyanin in ZSD extract (mg/g); [(A_520_-A_700_)] _pH_ _1.0_ represents the difference in absorbance values between the pH 1.0 buffer sample solution at 520 nm and 700 nm; [(A_520_-A_700_) _pH_ _4.5_] represents the absorbance difference for buffer solution (pH 4.5) between 520 nm and 700 nm; 449.2 is the molecular weight of cyanidin-3-glucoside; DF is the dilution factor; ε = 26,900 (molar extinction coefficient of cyanidin-3-glucoside, L·(mol·cm^−1^)); L is the path length, which is set to 1 cm.

### 2.5 Preparation of the pH-sensitive films

The chitosan/gelatin (CG) composite film was prepared following the method described by Yan et al. (2021). About 1.8 g of chitosan was dissolved in 90 mL of 2% (*v*/v) aqueous acetic acid solution and 0.2 g of gelatin in 10 mL of distilled water. The solutions were mixed together, and 0.2 g of glycerol was added to the mixture. Then, 0, 10, 15, and 20% ZSD extract (based on the polymer matrix) was added and stirred using a magnetic stirrer until well mixed, resulting in a film-forming solution. The CG composite film with 0, 10, 15, and 20% ZSD extract were named as follows: CG film, CG-ZSD10 film, CG-ZSD15 film, and CG-ZSD20 film.

### 2.6 Moisture content, thickness, and mechanical property

The moisture content was determined by drying the sample to a constant weight at 105 °C. The moisture content was calculated using formula (4):

(4) $$\begin{array}{c} \mathrm{MC}\left(\%\right)=\frac{W_{1}-W_{2}}{W_{1}}\times 100 \end{array}$$

where MC is the moisture content (%); W_1_ is the initial weight (g); W_2_ is the final weight (g).

The thickness was measured using a micrometer with an accuracy of 0.01 mm. Measurements were taken at four random locations on each film, and the average value was calculated.

According to the ASTM Standard Method, the film was cut into dimensions of 15 mm × 100 mm. The mechanical property including elongation at break (EB), tensile strength (TS), and Young's modulus (YM) were determined using a TA texturing device, with a tensile speed set at 10 mm/min (Chen et al., 2023).

### 2.7 Scanning electronic microscopy (SEM)

Prior to the analysis, the films were coated with a thin layer of gold, approximately 10 nm in thickness. The surface and cross-section micromorphology of film was observed using field emission scanning electron microscopy (VEGA3 TESCAN, TESKEN Co., Ltd., Czech) (Huang et al., 2023).

### 2.8 Fourier transform infrared (FTIR)

FTIR analysis was conducted using ALPHA infrared spectrometer (Bruker, Germany) in the range of 4000–400 cm^−1^. The spectra were obtained at 4 cm^−1^ resolution (Yong & Liu, 2020).

### 2.9 Thermogravimetric analysis (TGA)

TGA analysis of film was performed using the method outlined by Dong et al. (2019). The temperature was ramped from 30 °C to 600 °C, at 20 °C/min nitrogen flow rate.

### 2.10 Differential scanning calorimetry (DSC)

About 10 mg of film sample was placed in a hermetic pan and heated from 20 °C to 200 °C at a rate of 10 °C/min, while an empty hermetic pan was used as a control (Kumar et al., 2019).

### 2.11 X-ray diffraction (XRD)

Cu K-α radiation was generated using an X-ray Diffractometer (EMPYREAN Multifunctional, Malvern-Panalytical, UK) at 40 kV and 40 mA. The XRD spectra of film were analyzed by scanning at a speed of 2°/min and an angle range of 5°–80° (Parveen et al., 2024).

### 2.12 Light barrier ability

The film was analyzed using a dual-beam UV–vis spectrophotometer at 200–800 nm (Xu et al., 2022). An empty cuvette was used as the control.

### 2.13 Film color

The L, a, and b value of film was determined using a WSC-S colorimeter with D6 as the light source. The color difference value (∆E) was determined using formula (1).

### 2.14 pH- and ammonia-sensitivities of film

The film was cut into 2 cm × 2 cm square, immersed into buffer solution (pH = 1–14) for 5 min, and the color changes of film were photographed. The ammonia sensitivity of film was evaluated according to the procedure described by Jiang et al. (2023).

### 2.15 pH value, TVB-N value, and microbial analysis

For the fish freshness indication test, the CG-ZSD15 film was selected. The indicator label was made from CG-ZSD15 film and designed with different color according to fish freshness (‘Fresh’, ‘S-Fresh’, and ‘Putrid’). The *Pelteobagrus fulvidraco* fish was placed in a transparent container, and the indicator label was attached to the inner upper side of container. The container was sealed and stored at 4 ± 1 °C for 6 days. The color change of indicator label was photographed once a day, the color parameter (L, a, and b value) was recorded, and ∆E value was determined using formula (1).

The pH value, sensory quality, and total volatile basic nitrogen (TVB-N) value of fish were evaluated according to the Chinese standards GB 5009–2016 and GB 2733–2015. The total viable count (TVC) of fish was carried out using Chinese Standard GB 4789–2022 (Chen et al., 2023).

### 2.16 Statistical analysis

The data were analyzed utilizing software SPSS 27.0 version. One-way ANOVA was performed using Duncan test. The significance level was set at *p* < 0.05.

## 3 Results and discussion

### 3.1 Color change of ZSD extract

**Fig. 1:**
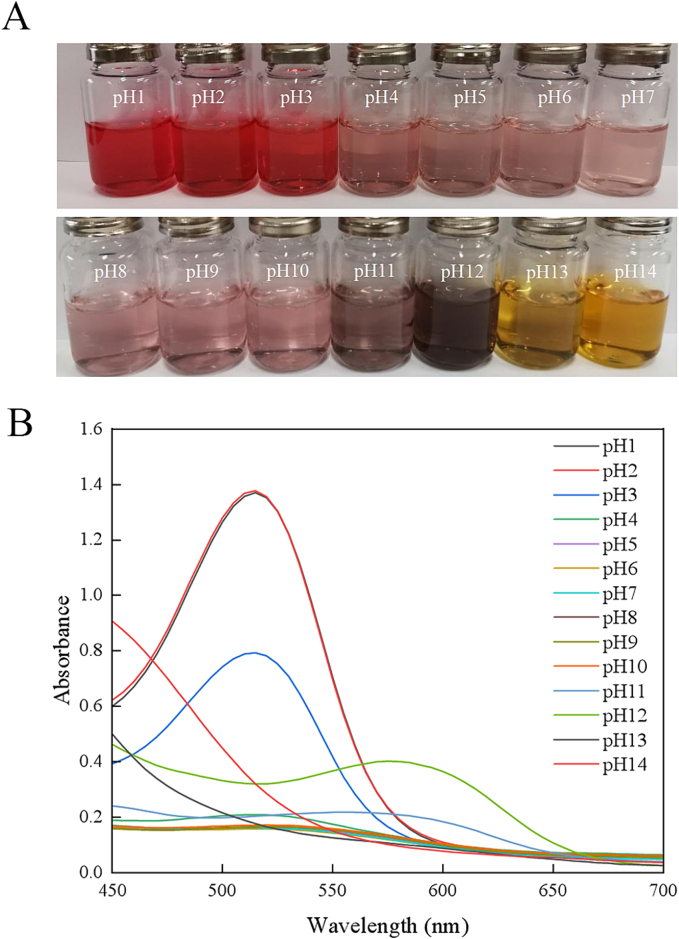
(A) The color change and (B) UV–vis spectra of ZSD extract in different buffer solutions (pH = 1–14).

The color variation of ZSD extract (pH = 1–14) was depicted in Fig. 1A. The anthocyanin content of ZSD extract was 228.8 mg/L. The main component of ZSD extract is flavonoid compound and anthocyanin. The anthocyanin of ZSD extract is red pigment and mainly contains peonidin-3-rhamnoside and malvidin-3-galactoside (Liu et al., 2023).

As illustrated in Fig. 1A, the color of anthocyanin isolated from ZSD changed from red (pH 1–3) to light pink (pH 4–10). As pH value increased from 11 to 14, the color changed from gray to dark gray and then to yellow. The color change might be attributed to the changes in the chemical structure of anthocyanin, as demonstrated in Fig. 1B. The maximum absorption peak occurred at 515 nm. However, the absorption peak shifted to 585 nm, with the gradual increase in pH value. Under high acidic condition, the yellow coloration was might be due to the formation of flavylium cation. The gray coloration was a result of the formation of methanol pseudobase in a weak acidic environment (Yong & Liu, 2020). Conversely, under alkaline environment, the coloration was due to the formation of chalcone, resulting in a yellow hue (Zheng et al., 2023). The observed pH-dependent color change in ZSD extract was aligned with the finding of Yan et al. (2021), who confirmed the viability of coffee bark extract into polymer matrix as a natural indicator.

### 3.2 UV–vis light transmittance

**Fig. 2:**
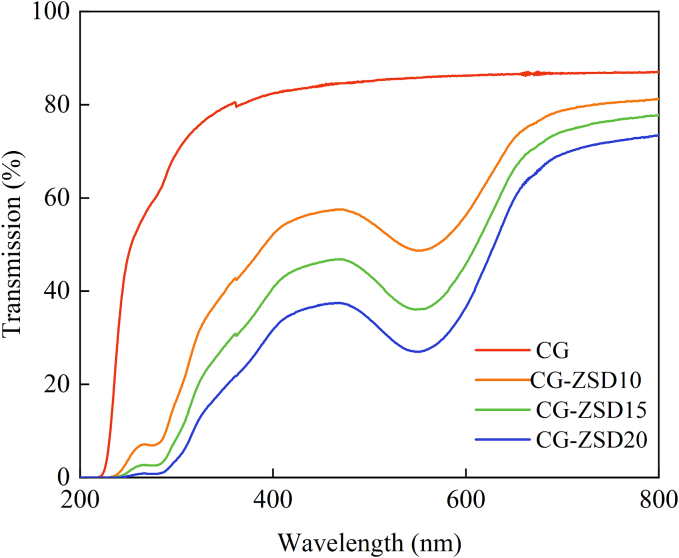
The UV–vis light transmittance of CG film and CG-ZSD composite films.

During food storage process, both visible light and ultraviolet would cause damage on food product, resulting in the loss of nutritional component. The use of food packaging with excellent UV–vis light barrier property is critical for maintaining food quality and extending its shelf life (Li et al., 2022). The UV transmittance of film at 200–800 nm was illustrated in Fig. 2. In the UV region (200–380 nm), the CG film exhibited higher UV transmittance compared to CG-ZSD film. The UV transmittance of CG film at 380 nm was 81.1%, while that of CG-ZSD film decreased from 46.8% to 26.1%. ZSD extract could significantly (*p* < 0.05) reduce the UV transmittance of CG-ZSD composite film. In the visible light region (380–780 nm), the visible light transmittance of CG film was also higher than that of CG-ZSD composite film. This might be because of the selective light absorption by anthocyanin (Merz et al., 2020). Recent studies also reported a decrease in UV–vis light transmittance after the incorporation of natural chromophores, such as *Oxalis triangularis* extract, *Syzygium cumini* anthocyanin, and red barberry anthocyanin (Jiang et al., 2023; Merz et al., 2020). Low UV–vis light transmittance indicated high opacity and barrier ability of film. This showed that the CG-ZSD composite film exhibited excellent light-blocking performance, which effectively protected food products from deterioration caused by photooxidation (Sani et al., 2021).

### 3.3 Thickness, moisture content, mechanical property, and color of film

The thickness, moisture content, and mechanical property of CG-ZSD composite film were provided in Table 1. From Table 1, it could be observed that there was no significant (*p* > 0.05) difference in moisture content among film samples. The addition of ZSD extract resulted in a gradual increase in the thickness of film. There was no significant (*p* > 0.05) difference in thickness between CG, CG-ZSD10, and CG-ZSD15 film. However, the addition of 20% ZSD extract led to a significantly (*p* < 0.05) higher thickness when compared to other films. This might be due to the strong hydrogen bonding between anthocyanin and polymer matrix (Chen et al., 2023). The appropriate amount of ZSD extract (< 20%) was well dispersed into the polymer matrix, leading to a more compact structure of film. Nevertheless, film thickness should not be the sole criterion for evaluating the film, but should be considered in conjunction with other properties for a comprehensive assessment (Xu et al., 2022).

**Table 1:** Physical characteristics including thickness, moisture content, color, and mechanical property of films (The CG film, CG-ZSD10 film, CG-ZSD15 film, and CG-ZSD20 film contain 0, 10, 15, and 20% ZSD extraction, respectively).

Film	CG	CG-ZSD10	CG-ZSD15	CG-ZSD20
L	91.1 ± 0.66^d^	61.5 ± 0.74^c^	58.8 ± 0.36^b^	52.1 ± 0.43^a^
a	−1.29 ± 0.14^a^	13.4 ± 0.49^c^	12.7 ± 0.38^b^	14.6 ± 0.36^d^
b	5.50 ± 0.23^d^	−2.40 ± 0.28^c^	−3.73 ± 0.23^b^	−4.41 ± 0.50^a^
ΔE	1.87 ± 0.26^a^	33.7 ± 0.76^b^	36.0 ± 0.29^c^	42.9 ± 0.43^d^
Color				
Moisture content (%)	15.8 ± 0.68^a^	15.3 ± 0.25^a^	15.1 ± 0.84^a^	15.6 ± 0.04^a^
Thickness (μm)	51.3 ± 0.50^a^	53.8 ± 5.25^a^	58.3 ± 8.46^a^	74.3 ± 5.85^b^
Elongation at break (%)	1.42 ± 0.37^a^	3.00 ± 0.77^b^	3.91 ± 0.69^b^	6.33 ± 0.62^c^
Tensile strength (MPa)	28.7 ± 0.51^d^	25.8 ± 0.41^c^	21.1 ± 0.47^b^	20.0 ± 0.58^a^
Young's modulus (MPa)	2009 ± 59.4^c^	1373 ± 67.0^b^	1316 ± 52.6^b^	1129 ± 66.0^a^

Note: All data were shown as mean ± standard deviation. Data with different letters indicated significant differences (*p* < 0.05).

The EB value is an indicator of film flexibility, while the TS value represents its mechanical resistance. According to Table 1, the addition of ZSD extract resulted in an increase in EB value from 1.42% to 6.33%. There was a significant (*p* < 0.05) difference in EB value between CG film and CG-ZSD films. The anthocyanin molecule weakened the intermolecular forces between neighboring macromolecules and enhanced the free-motion volume of the molecular chains, thereby improving the flexibility of film (Xu et al., 2022).

There was significant (*p* < 0.05) difference in TS value among film samples. When the content of ZSD extract increased from 0% to 20%, TS value decreased from 28.7 MPa to 20.0 MPa, and YM value decreased from 2009 MPa to 1129 MPa. This reduction might be attributed to the formation of hydrogen bonds between anthocyanin and polymer matrix, which leads to molecular migration and perturbation of solid structure (Anugrah et al., 2023).

The color variation of film was also presented in Table 1. The CG film, which did not contain ZSD extract, was nearly transparent and appeared light white. However, ZSD extract significantly (*p* < 0.05) reduced L value, resulting in decreased transparency and a purple color in the CG-ZSD films. With the increase in ZSD content, the CG-ZSD composite films exhibited a significant (*p* < 0.05) decrease in b value, while ΔE value significantly (*p* < 0.05) increased. ΔE value for pure CG film was only 1.87 ± 0.26, and that for CG-ZSD10, CG-ZSD15, and CG-ZSD20 film sharply increased to 33.7 ± 0.76, 36.0 ± 0.29, and 42.9 ± 0.43, about 18, 19, and 23 times higher than that of pure CG film, respectively. There was significant (*p* < 0.05) difference among the CG-ZSD films. Higher ΔE value indicated good visual color variability and allowed the films to be visually distinguished (Pourjavaher et al., 2017). This showed that the content of pH sensitive dye had an important role in color efficiency of pH indicator film (Chen et al., 2023).

### 3.4 SEM

**Fig. 3:**
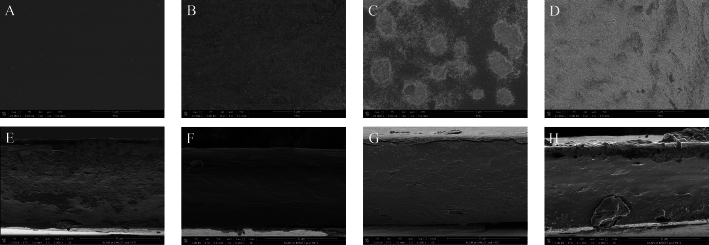
The surface images of (A) CG film, (B) CG-ZSD10 film, (C) CG-ZSD15 film, and (D) CG-ZSD20 film (24,000× magnification), and the cross-section images of (E) CG film, (F) CG-ZSD10 film, (G) CG-ZSD15 film, and (H) CG-ZSD20 film (6000× magnification).

The surface and cross-section morphology of CG, CG-ZSD10, CG-ZSD15, and CG-ZSD20 film were depicted in Fig. 3. As could be seen from Fig. 3A, the CG film illustrated a wrinkle-free and smooth surface. However, ZSD extract significantly influenced the film surface, as shown in Fig. 3B-D. The CG-ZSD composite film exhibited visible adherent on surface, resulting in a rougher texture. This change was more pronounced when the ZSD extract content was beyond 15%. Fig. 3 *E*-H displayed the cross-section images of film. There were some cracks and voids in the CG and CG-ZSD20 film. However, the cross-section images of CG-ZSD10 and CG-ZSD15 film were flatter than that of CG and CG-ZSD20 film. This indicated that appropriate amount of ZSD anthocyanin could improve the compatibility between chitosan and gelatin. An excess addition of anthocyanin would affect the compatibility between matrix with some signs of separation or cracks. This observation was similar to the result reported by other researches (Huang et al., 2023).

### 3.5 FTIR

**Fig. 4:**
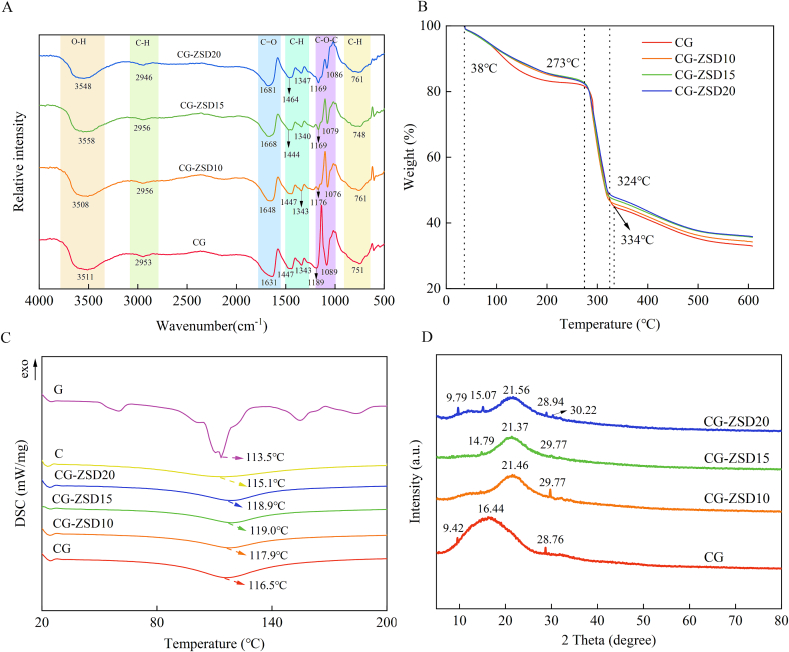
(A) Fourier transform infrared spectra, (B) Thermogravimetric analysis, (C) Differential scanning calorimetry, and (D) X-ray Diffraction spectra of the CG film and CG-ZSD composite films.

The FTIR analysis is conducted to examine the chemical structure of substance and the chemical reactions occurring between different materials (Yan et al., 2021). The FTIR spectra were shown in Fig. 4A. The appearance of characteristic absorption peak at 3558–3508 cm^−1^ is attributed to the stretching of O—H in alcohols and phenols (Khanjanzadeh & Park, 2021). Additionally, the characteristic absorption peak at 2956–2946 cm^−1^ is mainly related to the stretching of C—H bonds (Yu et al., 2018). The stretching of the amide I-chitosan C=O bond leads to characteristic absorption peaks at 1681–1631 cm^−1^ (Huang et al., 2020). Furthermore, the characteristic absorption peak at 1464–1340 cm^−1^ corresponds to the C—H in-plane bending vibration (Ezati et al., 2020). The characteristic absorption peak at 1189–1076 cm^−1^ is associated with the stretching of C-O-C bonds (Merz et al., 2020; Sani et al., 2021). Lastly, the characteristic absorption peak at 761–751 cm^−1^ is related to the C—H out-of-plane bending in the benzene ring (Chen et al., 2018).

The addition of ZSD extract resulted in slight changes in the FTIR spectral peaks of composite. With the addition of ZSD extract, the absorption peak for C=O bond shifted from 1631 cm^−1^ (CG film) to 1681 cm^−1^ (CG-ZSD20 film), and the C—H in-plane bending vibration shifted from 1447 cm^−1^ (CG film) to 1464 cm^−1^ (CG-ZSD20 film), and the peak for C-O-C bonds became sharper. However, no new peaks appeared in the spectra of the CG-ZSD films compared to the CG film, which indicated that no covalent bond formed in ZSD and polymer matrix. Thus, the incorporation of ZSD does not back change the chemical structures of CS and GL polymer. In addition, the O—H absorption peak for CG-ZSD film shifted to lower wave compared to the CG film, which indicated that the increase of hydrogen bonding between molecules could enhance the intermolecular compatibility (Syafiq et al., 2021).

### 3.6 TGA

The TGA analysis could reveal the thermal stability of film and the TGA image was illustrated in Fig. 4B (Hazarika et al., 2023). As shown in the TGA thermogram, the CG-ZSD composite film was decomposed through three-step thermal decomposition. At the first stage, the temperature ranged from 38 °C to 273 °C, and the weight loss was 16.5%. The weight loss at stage 1 might be due to the evaporation of glycerol and water from film (Dong et al., 2019). At the second stage (280–324 °C), the weight loss for CG-ZSD composite film was about 32.5%. However, the endpoint temperature for CG film was 334 °C. The addition of ZSD extract did not significantly affect the weight loss at the first and second stage.

At the third stage, spanning from 324 °C to 600 °C, the weight loss was 13.8%. This was due to the breakdown of chitosan and gelatin matrix (Kit & Kahyaoglu, 2024). As could be seen from Fig. 4B, at this stage, the weight loss of composite film gradually decreased with the increase in ZSD extract content. However, irrespective of the stages, the weight loss of the CG film was the highest. The addition of ZSD extract slightly delayed each step of the thermal degradation of CG-ZSD composite film (Dong et al., 2019). The heat-stable phenolic compound of ZSD extract is related to the improved thermal stability of the color indicator films (Sani et al., 2021). This was also accordance with the difference in their crystallinity (Roy & Rhim, 2020).

### 3.7 DSC

The DSC curve of chitosan, gelatin, and composite film was shown in Fig. 4C, along with the melting temperature (T_m_). The thermal stability of films was also evaluated by DSC analysis. The T_m_ value exhibited a broad heat absorption peak in the range of 100–120 °C, which might be attributed to the evaporation of adsorbed water. The T_m_ of chitosan and gelatin was 115.1 °C and 113.5 °C, respectively. Moreover, the T_m_ value of composite film gradually increased with the increase of ZSD extract, which indicated that the addition of ZSD extract improved the thermal stability of composite to some extent. However, when the ZSD addition was 15% and 20%, the T_m_ value was almost indistinguishable from each other. The result of the DSC analysis agreed with the results previously reported by Kumar et al. (2019).

### 3.8 XRD

The effect of anthocyanin from ZSD extract on the crystallinity of the films was characterized by XRD analysis, as depicted in Fig. 4D. The CG film exhibited a pronounced broad diffraction peak centered at 2θ = 16.44°, alongside minor peaks observed at 2θ = 9.42° and 2θ = 28.76°. These findings suggested that the addition of gelatin had resulted in a reduction in the crystalline nature of chitosan, corroborating with previous studies that have documented the presence of intense peaks in pure chitosan film, which was indicative of the crystal composition (Parveen et al., 2024). The film containing 10%, 15%, and 20% ZSD extract exhibited broad diffraction peaks at 2θ angle of 21.46°, 21.37°, and 21.56°, respectively. These peaks were significantly less intense when compared to those of the CG film, indicating a further reduction in crystallinity with increasing ZSD extract content. As previously discussed, the mechanical property of film depended on the microcrystalline structure within the films. The addition of ZSD extract would result in a competition for hydrogen bonding between ZSD and polymer matrix (Jiang et al., 2023). This interaction might hinder the formation of inter-matrix hydrogen bonding, which in turn reduced the crystallinity of film (Chen et al., 2020). This also corroborated the findings of the reduction in the mechanical property of film upon the addition of ZSD extract (Table 1). Similar trend in the XRD pattern was also observed for other biopolymer films containing anthocyanin (Abdillah et al., 2022; Chen et al., 2020).

### 3.9 pH- and ammonia-sensitivities of films

**Fig. 5:**
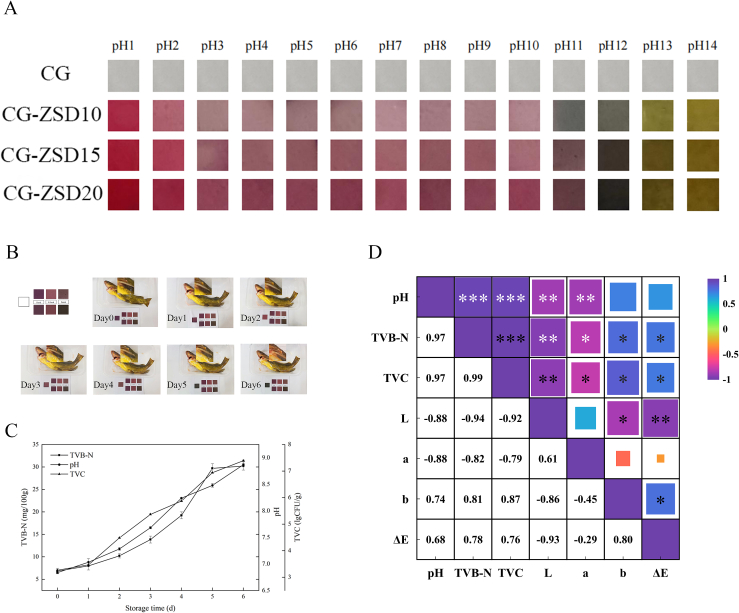
(A) The color change of CG film and CG-ZSD composite films in pH buffer solution (pH = 1–14), (B) the color change of CG-ZSD15 film during fish storage period, (C) pH value, total viable count, and total volatile basic nitrogen content during fish storage period, and (D) Pearson correlation between pH value, total volatile basic nitrogen content, total viable count, L, a, b, and ΔE value during fish storage period, where *p < 0.05, **p < 0.01, ***p < 0.001.

The pH sensitivity is an important characteristic for indicator film, as pH value is an indicator for food spoilage. The color change of film after immersion in buffer solution with pH value ranging from 1 to 14 was illustrated in Fig. 5A. The ZSD extract would affect pH sensitivity and color changes of the composite film. This was because of the structural changes of anthocyanin under different pH conditions. As could be seen from Fig. 5A, color change was more pronounced when 15% ZSD extract was added. Furthermore, a small amount of anthocyanin is insufficient to cause a significant color change, while excessive anthocyanin interferes with the film color change due to the inherent color of the extract itself. Therefore, the CG-ZSD15 film was selected in the subsequent fish detection test.

The color change of CG film and CG-ZSD films in an ammonia environment at different exposure time was presented in Table 2. The color of CG films remained essentially unchanged in the ammonia environment, and there was no significant (*p* > 0.05) difference in ΔE value. However, as the exposure time in the ammonia environment proceeded, there was a significant (*p* < 0.05) color change observed, transitioning from purple to green. The CG-ZSD films exhibited significant (*p* t 0.05) differences in L, a, b, and ∆E value, and there was a progressive change in film color from violet to green with increasing exposure time. The CG-ZSD20 film displayed the most noticeable color change visible to the naked eye, which further changed to dark green at peak sensitivity level. The color change and high sensitivity of CG-ZSD film in an ammonia environment were apparently attributed to the phenolic compounds in the anthocyanin from ZSD extract. Ammonium ions stimulate the creation of an alkaline condition on the surface of the color indicator film (NH_4_^+^), resulting from hydration and hydrolysis of ammonia vapor (NH_3_) inside the film (Abdillah et al., 2022). A relative short time (30 min) of rapid and high sensitive response to ammonia vapor confirmed the potential ability of the CG-ZSD indicator film to capture volatile nitrogen compound released from the spoiled aquatic product.

**Table 2:** The ammonia-sensitivities of CG, CG-ZSD10, CG-ZSD15, and CG-ZSD20 film during fish storage period (The CG film, CG-ZSD10 film, CG-ZSD15 film, and CG-ZSD20 film contain 0, 10, 15, and 20% ZSD extraction, respectively).

Film	Time /(min)	L	a	b	ΔE	color
CG	0	91.1 ± 0.66^d^	−1.29 ± 0.14^a^	5.50 ± 0.23^d^	1.87 ± 0.26^a^	
	10	91.2 ± 0.85^d^	−0.06 ± 0.39^a^	5.29 ± 0.62^c^	1.94 ± 0.42^a^	
	20	91.8 ± 0.47^d^	−0.67 ± 0.42^d^	4.70 ± 0.74^d^	1.51 ± 0.48^a^	
	30	91.5 ± 0.80^d^	−1.72 ± 0.12^c^	5.07 ± 0.50^d^	1.77 ± 0.25^a^	
CG- ZSD10	0	61.5 ± 0.74^c^	13.4 ± 0.49^c^	−2.40 ± 0.28^c^	33.6 ± 0.76^b^	
	10	64.7 ± 0.98^c^	6.00 ± 0.86^b^	1.05 ± 0.16^b^	27.7 ± 1.10^b^	
	20	65.3 ± 0.11^c^	−3.86 ± 0.94^bc^	1.04 ± 0.37^b^	26.3 ± 0.19^b^	
	30	62.0 ± 1.08^c^	−1.73 ± 0.26^c^	0.41 ± 0.17^c^	29.5 ± 1.07^b^	
CG- ZSD15	0	58.8 ± 0.36^b^	12.7 ± 0.49^b^	−3.73 ± 0.23^b^	36.0 ± 0.29^c^	
	10	60.0 ± 0.85^b^	−0.62 ± 0.83^a^	−0.33 ± 0.29^a^	31.6 ± 0.83^c^	
	20	61.8 ± 0.93^b^	−8.50 ± 0.60^b^	2.03 ± 0.26^c^	30.6 ± 1.02^c^	
	30	58.8 ± 0.53^b^	−7.59 ± 0.52^a^	−0.45 ± 0.36^b^	33.4 ± 0.48^c^	
CG- ZSD20	0	52.1 ± 0.43^a^	14.6 ± 0.36^d^	−4.41 ± 0.50^a^	42.9 ± 0.43^d^	
	10	50.3 ± 0.74^a^	10.6 ± 0.74^c^	1.52 ± 0.06^b^	42.6 ± 0.84^d^	
	20	54.2 ± 0.16^a^	−11.0 ± 0.43^a^	−2.29 ± 0.31^a^	38.9 ± 0.20^d^	
	30	47.1 ± 0.38^a^	−4.86 ± 0.39^b^	−2.64 ± 0.29^a^	44.8 ± 0.44^d^	

Note: All data were shown as mean ± standard deviation. Data with different letters indicated significant differences (*p* < 0.05).

### 3.10 Application of pH-sensitive film for fish freshness monitoring

The label design for CG-ZSD15 indicator film and the color change during fish preservation on day 0, 1, 2, 3, 4, 5, and 6 were depicted in Fig. 5B, and the data of color change were listed in Table 3. As time proceeding, the color of indicator label transitioned from purple to rosy red, then to brown, and eventually to dark gray. As the storage time extended, the L and a value exhibited a decreasing trend, while the b and ΔE values showed an increasing trend, which correlated with the pH response behavior of the indicator films. On the 0th day of storage, the film color was purple. By the fourth day, the film exhibited a dark brown color, and on the 6th day, it turned dark gray, indicating complete deterioration of the fish meat. The significance analysis confirmed that the L, a, b, and ΔE values were significantly (*p* < 0.05) different on the 0th day, 4th day (TVB-N limit), and 6th day (complete fish deterioration), which could be distinguished by naked eyes during fish storage period. The ΔE value also reflected the color change of film as a function of time.

**Table 3:** The color change of CG-ZSD15 film during fish storage period.

Storage time/(d)	L	a	b	ΔE	Color
0	58.8 ± 0.36^d^	12.7 ± 0.49^d^	−3.73 ± 0.23^a^	36.0 ± 0.29^a^	
1	52.9 ± 0.13^c^	20.1 ± 0.59^g^	−3.34 ± 0.76^a^	44.3 ± 0.29^b^	
2	54.1 ± 1.24^c^	17.1 ± 0.46^e^	−0.02 ± 0.83^b^	41.5 ± 1.38^c^	
3	48.3 ± 0.44^b^	18.8 ± 0.08^f^	2.48 ± 0.64^c^	47.3 ± 0.35^d^	
4	45.0 ± 0.49^a^	10.0 ± 0.32^c^	1.76 ± 0.13^c^	47.6 ± 0.56^d^	
5	45.1 ± 0.48^a^	4.93 ± 0.30^b^	2.32 ± 0.5^c^	46.6 ± 0.46^d^	
6	44.0 ± 1.00^a^	3.40 ± 0.23^a^	1.65 ± 0.32^c^	47.8 ± 1.03^d^	

Note: All data were shown as mean ± standard deviation. Data with different letters indicated significant differences (*p* < 0.05).

The changes in pH, TVB-N content, and TVC count of fish during storage period were illustrated in Fig. 5C. It could be observed from Fig. 5C that the pH value, TVB-N content, and TVC count exhibited a gradual increase from day 0 to day 6. According to Chinese standard GB 2707–2016 ‘Hygienic Standard for Fresh (Frozen) Meat’, the limit of TVB-N value for freshwater fish and shrimp is 20 mg/100 g, which could be judged that the meat is still in the fresh period (Chen et al., 2023). The TVB-N content reached 19.2 mg/100 g on day 4, almost reaching the permissible limit. After 6 days of storage, the fish sample emitted a strong foul odor, the texture of became smoother, and white mycelium was visible on the surface, indicating a state of deterioration. The pH and TVB-N content are closely associated with the microorganism growth, and are important indicators used to assess the freshness of aquatic product (Huang et al., 2020). During storage, the metabolism of microorganisms responsible for fish spoilage produces various volatile gases that slowly release to the headspace of the storage container (Wang et al., 2019). Over time, the CG-ZSD15 film absorbed the volatile gases, leading to an alkaline state, which, in turn, altered the structure of anthocyanin in the film. This structural change induced a color change in the film, enabling the detection of fish freshness based on color (Hazarika et al., 2023).

Some polymeric indicator films with natural anthocyanin were also studied to indicate fish freshness. Hazarika et al. (2023) reported that the cellulose nanofibers /cellulose acetate film with anthocyanin from *Melastoma Malabathricum* seed was highly sensitive to ammonia vapor and could be used as a label to indicate the freshness of fish. Yan et al. (2021) prepared a chitosan film with anthocyanin from butterfly pudding extract. The TVB-N content and pH value changes of tilapia fish were closely related to the visual color changes in film. The changes in pH and TVB-N content of fish were closely associated with the color change of CG-ZSD15 film, demonstrating the feasibility of using the film as an indicator label to differentiate the freshness of fish.

### 3.11 Correlation analysis

The Pearson correlation was analyzed to investigate the associations between pH, TVB-N content, and TVC with the L, a, b, and ΔE values of fish during storage, as depicted in Fig. 5D. The results indicated that there was a negative correlation between the pH of fish and the L (*r* = −0.88, *p* < 0.01) and a (r = −0.88, *p* < 0.01) values of the film. Additionally, there was a positive correlation between the pH of fish and the TVB-N content (*r* = 0.97, *p* < 0.001), TVC (r = 0.97, *p* < 0.01), b, and ΔE values. This can be attributed to the fact that, during storage, the TVC of fish meat gradually increased, leading to the continuous decomposition of proteins and nitrogenous substances in the fish meat under the influence of enzymes and bacteria. Consequently, the pH value and TVB-N content of fish increased, resulting in corresponding changes in the color indexes of the film (L, a, b, and ΔE) (Huang et al., 2014). These results suggest that the CG-ZSD15 coated film effectively indicates the degree of fish meat deterioration and serves as a suitable pH-sensitive film for assessing fish meat freshness.

## 4 Conclusion

In this study, a pH-sensitive film was developed for monitoring fish freshness using ZSD anthocyanin, chitosan, and gelatin. The incorporation of ZSD extract led to changes in the mechanical property, thickness and color of film, enhanced changes in its UV-blocking property, while reducing its water content. The SEM and FTIR results illustrated that a dense structure was formed between ZSD extract, chitosan, and gelatin matrix. The DSC and TGA results showed that ZSD extract could improve the thermal stability of film. The composite film also exhibited significant color changes at different pH value and demonstrated excellent sensitivity to ammonia. The application test revealed that the CG-ZSD15 film provided evident color response of fish spoilage.

Despite the lowered tensile strength and Young's modulus caused by the inclusion of ZSD extract, the composite film still outperformed most chitosan/gelatin films in these two aspects. This study introduces for the first time information regarding the stability and application of anthocyanins from *Zingiber striolatum* Diels as an alternative for food packaging. In the future, more researches are required to establish a more accurate indication relationship between indicator film and fish freshness, and the reduction in swelling property of film might expand the application prospect of anthocyanin intelligent composite films.

## Ethical guidelines

Ethics approval was not required for this research.

## CRediT authorship contribution statement

**Yuyue Qin:** Writing – review & editing, Validation, Supervision, Resources, Project administration, Funding acquisition, Conceptualization. **Yurou Wang:** Writing – review & editing, Methodology, Investigation, Data curation. **Zhenya Tang:** Writing – review & editing, Visualization, Supervision, Resources, Project administration, Funding acquisition, Conceptualization. **Kejun Chen:** Writing – original draft, Software. **Zhengxuan Wang:** Writing – review & editing, Resources. **Guiguang Cheng:** Methodology, Data curation. **Hai Chi:** Software, Investigation, Data curation. **Thanapop Soteyome:** Supervision, Conceptualization.

## Declaration of competing interest

The authors declare that they have no known competing financial interests or personal relationships that could have appeared to influence the work reported in this paper.

## Data Availability

Data will be made available on request.
